# Frequent Detection of HPV before and after Initiation of Antiretroviral Therapy among HIV/HSV-2 Co-Infected Women in Uganda

**DOI:** 10.1371/journal.pone.0055383

**Published:** 2013-01-29

**Authors:** Anne F. Rositch, Patti E. Gravitt, Aaron A. R. Tobian, Kevin Newell, Thomas C. Quinn, David Serwadda, Paschal Ssebbowa, Valerian Kiggundu, Ronald H. Gray, Steven J. Reynolds

**Affiliations:** 1 Department of Epidemiology, Johns Hopkins Bloomberg School of Public Health, Baltimore, Maryland, United States of America; 2 Department of Molecular Microbiology and Immunology, Johns Hopkins Bloomberg School of Public Health, Baltimore, Maryland, United States of America; 3 Perdana University Graduate School of Medicine, Serdang, Malaysia; 4 Department of Pathology, School of Medicine, Johns Hopkins University, Baltimore, Maryland, United States of America; 5 Clinical Research Directorate/Clinical Monitoring Research Program, SAIC-Frederick, Inc., Frederick National Laboratory for Cancer Research, Frederick, Maryland United States of America; 6 Rakai Health Sciences Program, Entebbe, Uganda; 7 Division of Intramural Research, National Institute of Allergy and Infectious Diseases, National Institutes of Health, Bethesda, Maryland, United States of America; 8 Department of Medicine, School of Medicine, Johns Hopkins University, Baltimore, Maryland, United States of America; Fundacion Huesped, Argentina

## Abstract

**Objectives:**

Most data on HPV and antiretroviral therapy (ART) come from high-resource countries with infrequent sampling for HPV pre- and post-ART initiation. Therefore, we examined the frequency of cervical HPV DNA detection among HIV/HSV-2 co-infected women followed monthly for 6 months both before and after initiation of ART in Rakai, Uganda.

**Methods:**

Linear Array was used to detect 37 HPV genotypes in self-collected cervicovaginal swabs from 96 women who initiated ART. Random-effects log-binomial regression was used to compare the prevalence of HPV detection in the pre- and post-ART periods and determine other potential risk factors, including CD4 counts and HIV viral load.

**Results:**

Nearly all women had detectable HPV in the 6 months preceding ART initiation (92%) and the cumulative prevalence remained high following initiation of therapy (90%). We found no effect of ART on monthly HPV DNA detection (prevalence ratio: 1.0; 95% confidence interval: 0.96, 1.08), regardless of immune reconstitution or HIV viral suppression. Older age and higher pre-ART CD4 counts were associated with a significantly lower risk of HPV DNA detection.

**Conclusions:**

ART did not impact HPV detection within 6 months of therapy initiation, highlighting the importance of continued and consistent screening, even after ART-initiation and immune reconstitution.

## Introduction

Oncogenic human papillomavirus (HR-HPV) is one of the most prevalent sexually transmitted infections (STI) worldwide and is a necessary cause of invasive cervical cancer [1], [2], [3]. Up to 90% of HIV-infected individuals are co-infected with HPV [4]. The incidence, prevalence, persistence, and recurrence/reactivation of HPV infection are greater among HIV-positive and immunosuppressed women than HIV-negative women [5], [6], [7], [8], [9]. This translates into an increased risk of cervical precancerous lesions and progression to invasive cervical cancer among HIV-positive women [6], [10], [11].

Given the strong association between HIV-related immunosuppression and detection of HPV, one could expect that women initiating or receiving antiretroviral therapy (ART) would have a lower risk of HPV infection due to improvements in immune function. However, previous studies of pre- and post-ART initiation among adolescent girls [12] and adult women [13] from the US, and studies comparing women receiving ART to those who are not [12], [14] have shown little decline in HPV outcomes over extended follow-up. However, the majority of data on HPV and antiretroviral therapy (ART) have come from high-resource countries and no studies with frequent sampling for HPV pre- and post-ART initiation have been conducted. In order to provide data from the developing world where the rates of HIV and HPV-associated anogenital cancers are disproportionately high, we examined the prevalence and frequency of cervical HPV DNA detection among 96 HIV-positive, HSV-2 co-infected women followed prospectively with monthly samples 6 months before and 6 months after initiation of ART in Rakai, Uganda.

## Materials and Methods

### Study design and population

HIV and HSV-2 co-infected individuals (N = 440) were enrolled in a randomized control trial of HSV-2 suppression to assess HIV disease progression in Rakai, Uganda, from May 2007 to November 2008 [15]. Briefly, individuals aged 18 years or older, with a CD4 count between 300 and 400 cells/µl were included; individuals with AIDS-defining illnesses or those currently receiving antiretroviral therapy (ART) were excluded. Individuals were randomly assigned to receive either placebo or 400 mg acyclovir twice daily for 24 months and were followed monthly until trial closure in October 2010. All individuals with documented genital ulcer disease (GUD) at enrollment or during the study were treated with acyclovir, regardless of trial arm. ART was initiated if women had a CD4 cell count that declined below 250 cells/µl or if women developed WHO stage 4 clinical disease.

At enrollment, a short interview was conducted to obtain information on sociodemographic characteristics. At enrollment and biannual study visits, a physical examination was conducted to diagnose GUD and collect blood for serologic testing. At monthly study visits, female participants were requested to provide a self-administered cervicovaginal swab. They were instructed to squat, insert a 20-cm Dacron or cotton-tipped swab and to rotate the swab high in the vaginal vault. After collection, swabs were stored in specimen transport medium (Digene Corporation, Gaithersburg, MD) and maintained at 4–10°C for less than 6 hours until frozen at −80°C for later HSV-2 and HPV DNA detection. There were 96 women who provided up to 6 monthly self-swabs in the 6 months immediately preceding initiation of ART and 6 monthly swabs immediately following ART initiation.

### Ethics statement

All study participants provided written informed consent. The trial was approved by the Uganda National Council for Science and Technology (Kampala, Uganda), Uganda Virus Research Institute Science and Ethics Committee, and the NIAID Intramural Institutional Review Board. The trial was registered with Clinical.Trials.Gov numbers NCT00405821.

### Laboratory Testing

At enrollment, HSV-2 serostatus was determined using Focus HerpeSelect-2 EIA (Cypress, CA, USA) and HIV-1 serostatus was determined using the Vironostika HIV-1 (Charlotte, NC, USA) and Organon Teknika (Cambridge Biotech, Worcester, MA, USA) enzyme immunoassays (EIA). Discordant EIAs were confirmed with HIV-1 western blot (Bio-Merieux-Vitek, St. Louis, MS, USA). The FACSCalibur (Becton Dickenson, Franklin Lakes, NJ, USA) was used to determine CD4 cell counts per µL and the Roche Monitor v1.5 assay (Indianapolis, IN, USA) was used to determine plasma HIV viral load copies/mL. Based on these tests, we defined immune reconstitution as a CD4 count increase of 50 or more cells/µL from the pre- to post-ART measurement [16] and defined HIV virologic suppression as undetectable HIV-1 viral load (<400 copies/mL) in the post-ART period.

Detection and genotyping of HPV DNA from self-administered cervicovaginal swabs was conducted at Johns Hopkins University in Baltimore, MD. DNA was extracted using the QIAamp DNA Blood Kit (Qiagen, Valencia, CA, USA) according to manufacturer's instructions with modification [17]. An 8 µl aliquot of extracted DNA was tested using the Roche HPV Linear Array PCR-based assay (Indianapolis, IN, USA). Detection of the presence of human DNA by beta-globin-specific polymerase chain reaction (PCR) is a component of the LA assay, and was used to determine sample adequacy. The HPV Linear Array is based on the PGMY09/11 PCR primer system that allows for high efficiency amplification of 37 distinct HPV genotypes [18], [19]. For this analysis, HPV types 16, 18, 31, 33, 35, 39, 45, 51, 52, 56, 58, 59, 66, and 68 were classified as high-risk (HR) HPV types.

### Statistical Analysis

Characteristics of the 96 women were summarized. Longitudinal detection of HPV DNA was assessed in the pre-ART, post-ART and pre-compared to post-ART initiation periods. Descriptive analyses, including numbers and percentages, means (standard deviations) and medians (inter-quartile ranges (IQR)), were used to examine the prevalence, average number of HPV types, and cumulative number of HPV types detected, as well as to compare changes in HPV DNA detection in the pre-ART versus post-ART periods overall and by changes in CD4 counts.

Random effects log-binomial regression models with a compound symmetry correlation structure were used to estimate prevalence ratios (PR) and 95% confidence intervals (CI) for the association between potential risk factors for HPV DNA detection over time in the pre- and post-ART periods. These models were also used to compare the prevalence of HPV DNA detection in the pre-ART compared to post-ART periods, where women served as their own control. The random effects models account for the correlation within women due to repeat observations across time. CD4 counts and HIV viral load measurements were analyzed using both time-invariant pre-ART measurements and time-varying measurements based on the corresponding pre- and post-ART period values. The pre-ART period CD4 count and HIV viral load measurements were the last measurement prior to ART initiation and the post-ART CD4 count and HIV viral load measures were defined as the first measurement within 2–9 months following ART initiation. All analyses were conducted in SAS version 9.3 (Cary, NC, USA).

## Results

### Population characteristics

The 96 women provided data on HPV DNA detection for 1076 out of 1152 possible study visits (93%). The median age of women in the study population at baseline was 35 years (IQR: 31–44) (Table 1). Forty-five percent of women were taking acyclovir and 55% of women were randomized to the placebo arm. All women in this sub-study initiated antiretroviral therapy, with 84% of women receiving AZT/3TC/NVP and 16% receiving non-AZT/3TC/NVP combination therapy. Within 6 month prior to initiating ART, the median CD4 count was 216.5 cells per µL (IQR: 178.0–235.5) and the median log_10_ HIV viral load was 4.9 (IQR: 4.3, 5.4). Within 9 months after initiating ART, the median CD4 count was 307.0 cell per µL (IQR: 162.0–377.0). The majority of women had immune reconstitution (73%) and HIV virologic suppression (96%).

**Table 1:** Characteristics of the female study population (N = 96).

	Population characteristics
	Median (IQR) or N (%)
**Baseline age (years)**	34.5 (30.5, 44.0)
**Trial arm**	
Placebo	53 (55.2%)
Acyclovir	43 (44.8%)
**Pre-ART period CD4 count (cells/µL)**	216.5 (178.0, 235.5)
**Pre-ART period HIV viral load (log_10_ copies/mL)**	4.9 (4.3, 5.4)
**ART regimen initiated**	
AZT/3TC/NVP	81 (84.4%)
CBV/EFV	5 (5.2%)
D4T/3TC/NVP	6 (6.3%)
TDF/3TC/NVP	3 (3.1%)
TVD/EFV	1 (1.0%)
**Post-ART period CD4 count (cells/µL)**	307.0 (162.0, 377.0)
**Post-ART immune reconstitution** a	
No	24 (26.7%)
Yes	66 (73.3%)
**Post-ART virologic suppression** b	
No	4 (4.5%)
Yes	85 (95.5%)

Abbreviations: interquartile range (IQR); number (n); percent (%); antiretroviral therapy (ART); zidovudine (AZT); lamivudine (3TC); nevirapine (NVP); Combivir (CBV); efavirenz (EFV); stavudine (D4T); tenofovir (TDF); Truvada (TVD).

a Immune reconstitution: CD4 count increase of 50 or more cells/µL from the pre- to post-ART measurement. Six women did not have follow-up CD4 counts in the 2–9 month post-ART window so immune reconstitution could not be calculated.

b HIV virologic suppression: undetectable HIV-1 viral load (<400 copies/mL) in the post-ART period. Seven women did not have follow-up CD4 counts in the 2–9 month post-ART window so immune reconstitution could not be calculated.

### Patterns of monthly HPV DNA detection pre- and post-ART initiation

**Figure 1:**
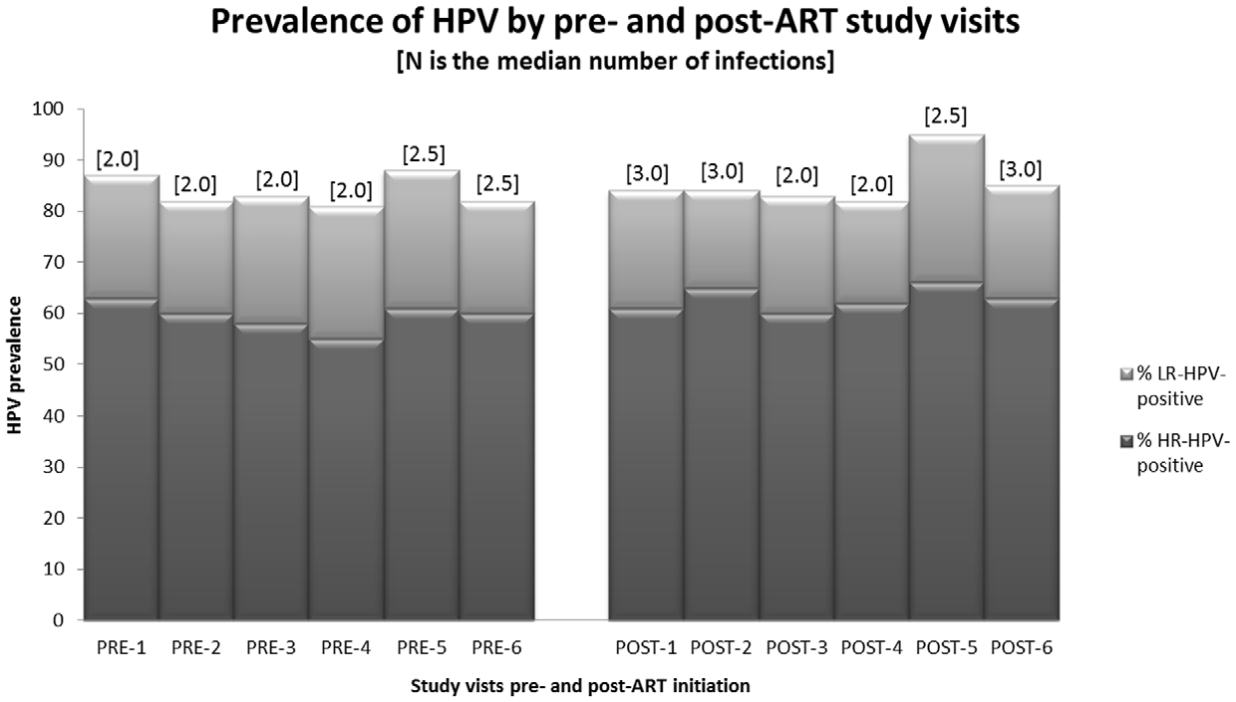
Pattern of high- and low-risk monthly HPV detection and median number of HPV [in brackets] types during the pre- and post-ART initiation periods. Abbreviations: infection with only low-risk human papillomavirus types (LR-HPV); high-risk human papillomavirus types alone or with LR-HPV types (HR-HPV).

The cumulative prevalence of any-HPV DNA detection over one year of monthly samples was 92% and was 76% for HR-HPV detection. In the pre-ART period, 8 women had no detectable HPV and these women remained HPV negative in the post-ART period (Table 2). Only low-risk (LR) HPV types were detected over the pre-ART period in 22 women, most of whom remained low-risk detectable in the post-ART period (68%). Of the 66 women with HR-HPV types (with or without LR-HPV types) during the pre-ART period, most remained HR-HPV detectable (94%). There was little variation in the prevalence of detection of LR and HR-HPV types and little difference in the median numbers of infections across monthly study visits (Figure 1). The monthly HPV prevalence estimates before ART initiation ranged from 81–88% and after ART initiation ranged from 82–95%. In addition, the cumulative prevalence of HPV DNA detection did not change from the 6 months prior to ART initiation (92% any-HPV and 69% HR-HPV) as compared to the 6 months after ART initiation (90% any-HPV and 72% HR-HPV).

**Table 2:** Cross-classification of cumulative HPV infection status in the 6 months before compared to after ART initiation.

	Post-ART HPV status			
Pre-ART HPV status	HPV-negative	LR-only HPV	HR-HPV	*Total*
HPV-negative	8 (100%)	0 (0%)	0 (0%)	*8 (8%)*
LR-only HPV	0 (0%)	15 (68%)	7 (32%)	*22 (23%)*
HR-HPV	2 (3%)	2 (3%)	62 (94%)	*66 (69%)*
*Total*	*10 (10%)*	*17 (18%)*	*69 (72%)*	

Abbreviations: infection with only low-risk human papillomavirus types (LR-HPV); high-risk human papillomavirus types alone or with LR-HPV types (HR-HPV).

### Changes in HPV DNA detection by post-ART changes in CD4 counts

Regardless of the change in CD4 count from the pre- to post-ART period, there were no changes in the median proportion of HPV-positive visits, median number of HPV types per visit, or median number of independent HPV types from the pre- to post-ART periods (Table 3). When the mean was used, data suggest a trend of fewer HPV positive visits (p = 0.04), but no change in the number of HPV types per visit (p = 0.34) or cumulative number of independent HPV types (p = 0.60) in the post-ART period as compared to the pre-ART period as the CD4 count increased. The slight differences between the medians and means highlight the skew of these distributions. Similar patterns of no to minor changes in HPV infection by changes in CD4 counts were also observed when restricted to HR-HPV types only (data not shown).

**Table 3:** Changes in HPV detection by changes in pre-vs. post-ART CD4 counts.

		Change in the proportion of HPV-positive visits^a^		Change in the number of HPV types per visit^a^		Change in the cumulative number of types^a^	
Pre- to post-ART CD4 count change	N	Mean(STD)	Median (IQR)	Mean(STD)	Median (IQR)	Mean(STD)	Median (IQR)
Change ≤0^b^	12	8.3 (19.5)	0 (0, 16.7)	1.2 (3.4)	0.3 (−0.4, 1.1)	0 (2.7)	0 (−1.5, 2.0)
Change 1–200	62	−0.3 (15.2)	0 (0, 0)	0.7 (2.3)	0.2 (−0.3, 1.1)	−0.2 (2.1)	0 (−1, 0)
Change >200	16	−7.9 (18.0)	0 (0, 0)	−0.1 (1.4)	0 (−1.4, 0.2)	−0.8 (1.8)	−0.5 (−2, 0)

Abbreviations: human papillomavirus (HPV); number (N); standard deviation (STD); interquartile range (IQR); antiretroviral therapy (ART).

a Change less than 0 implies that there was a decrease in the variable from the pre- to post-ART period.

b Change ≤0 implies that the CD4 cell count taken within 2–9 months after ART initiation was lower than the CD4 count taken within 6 months before ART initiation.

### ART initiation and other determinants of HPV DNA detection

Using regression models that account for the correlation within women, HPV DNA detection decreased with increasing age, regardless of study period (Table 4). Cumulative prevalence ranged from 98% among women age 20–29 years to 56% among women 50 years and older, with a prevalence ratio of 0.58 (95% CI: 0.33, 1.00). In addition, the prevalence of HPV detection decreased with increasing pre-ART CD4 count, with an estimated 14% reduction in HPV per 100 CD4 count increase (PR: 0.86; 95% CI: 0.78, 0.93). There was no association between HPV detection and time-varying CD4 count (data not shown). There were no associations between pre-ART HIV viral load or time-varying viral load and HPV detection. The prevalence of HPV detection during the late pre-ART period (study visits 4–6 immediately before ART initiation) was slightly greater than HPV detection during the early pre-ART initiation period (study visits 1–3 pre-ART) (PR: 1.07; 95% CI: 1.02, 1.12). With regards to the main effect of ART initiation, there was no difference in HPV 1-3 months post-ART (PR: 1.01; 95% CI: 0.96, 1.03) or 4–6 months post-ART (PR: 1.04; 95% CI: 0.98, 1.10) as compared to 1–3 month pre-ART HPV detection.

**Table 4:** Prevalence ratios for detection of any type HPV per visit per women in the pre-ART, post-ART and pre-vs. post-ART periods.

	HPV detection	
	n/visits (%)	PR (95% CI) a
**Age (years)**		
20–29	186/190 (98%)	1
30–39	465/527 (88%)	0.90 (0.82, 0.97)
40–49	204/250 (82%)	0.83 (0.71, 0.98)
≥50	48/85 (56%)	0.58 (0.33, 1.00)
**Trial arm**		
Control	516/584 (88%)	1
Acyclovir	387/492 (79%)	0.89 (0.76, 1.04)
**Pre-ART CD4 count (cells/µL)**		
per 100 count increase		0.86 (0.78, 0.93)
**Pre-ART HIV viral load (copies/mL)**		
<49,999	304/384 (79%)	1
50,000–199,999	294/330 (89%)	1.12 (0.94, 1.34)
200,000+	305/362 (84%)	1.07 (0.88, 1.29)
**ART regimen**		
None (pre-ART)	436/517 (84%)	1
Other	83/89 (93%)	1.08 (1.01, 1.16)
AZT/3TC/NVP	384/470 (82%)	0.98 (0.95, 1.03)
**ART initiation** b		
Early pre-ART	239/301 (79%)	1
Late pre-ART	197/216 (91%)	1.07 (1.02, 1.12)
Early post-ART	249/306 (81%)	1.01 (0.96, 1.08)
Late post-ART	218/253 (86%)	1.04 (0.98, 1.10)
**Immune reconstitution** c		
No	237/269 (88%)	1
Yes	610/747 (82%)	0.92 (0.80, 1.08)

Abbreviations: human papillomavirus (HPV); number (N), percent (%); prevalence ratio (PR); confidence interval (CI); antiretroviral therapy (ART); zidovudine (AZT); lamivudine (3TC); nevirapine (NVP); Combivir (CBV); efavirenz (EFV); stavudine (D4T); tenofovir (TDF); Truvada (TVD).

a Random effects log-binomial regression to estimate the prevalence ratio of HPV detection by covariates within and across study period, to account for repeated observations on the same women over time.

b Early pre-ART refers to 4–6 months before ART initiation and late pre-ART refers 1–3 months before initiation. Early post-ART refers to 1–3 months after initiation and late post-ART refers to 4–6 months after initiation. No significant interactions were found between ART initiation and other covariates (at p≤0.10).

c Immune reconstitution: CD4 count increase of 50 or more cells/µL from the pre- to post-ART measurement.

## Discussion

Nearly all HIV-positive women (92%) had detectable HPV infections in the 6 months preceding ART initiation and the cumulative prevalence remained high in the 6 months following initiation of therapy (90%). We found no effect of ART initiation on any- or HR-HPV prevalence overall and among women with immune reconstitution or HIV viral suppression. Older age and higher pre-ART CD4 counts were associated with a significantly lower risk of HPV DNA detection, whereas the late pre-ART compared to early pre-ART period was associated with slightly higher risk of HPV detection.

We examined a short but clinically relevant period of immune reconstitution with monthly samples in order to determine if, relative to previous studies with long testing intervals (e.g. 6 months), use of ART could successfully increase immunological control of HPV infection. Despite clinical markers indicating the majority of women experienced immune reconstitution, we observed no overall effect of ART on HPV detection. Consistent with previous studies using longer testing intervals and follow-up [12], [13], [14], [20], our findings suggest that while women did show a favorable systemic response to antiretroviral treatment, it is possible that the immune environment in the genital tract had not yet recovered sufficiently to gain control of localized infections such as HPV. It is possible that longer follow-up is required to observe a reduction in HPV detection, either by decreasing susceptibility to new HPV infection or by promoting control over existing infections after ART initiation [21]. However, on the population-level, most data show that the incidence of HPV-associated anogenital cancers has not decreased since the introduction of highly-active ART (HAART) [22], [23] and many, though not all [24], [25], [26], [27], cohort studies also report little or no effect of ART on incidence or regression of cervical precancer [13], [28], [29], [30], [31].

We observed a strong association between pre-ART CD4 count and HPV detection. However, time-varying CD4 count was not predictive of HPV detection. These findings highlight the dependence of genital HPV infection on the low, pre-ART CD4 counts even after ART-initiation, and are consistent with the fact that immune reconstitution was not associated with a decrease in HPV detection post-ART. It is important to note that we only had a single measurement of CD4 count in each study period, so counts were not concurrent with monthly HPV measurements and the exact timing of CD4 count measurement varied within each period. For example, it is likely that the increase in HPV detection in the late pre-ART as compared to early pre-ART period, as estimated by the frailty models, corresponds to the underlying sharp decline in CD4 counts that prompted initiation of therapy. A remaining gap in the literature is whether ART could improve control of and reduce the burden of HPV infection and associated precancer if it were initiated earlier in the course of HIV [32]. Ugandan national guidelines now recommend ART initiation at a CD4 count of 350 cells per µL or less and recent studies highlight several benefits of early ART initiation to both HIV-infected and uninfected partners [33], [34].

A potential limitation to our study is the fact that all women were co-infected with HSV-2 and half were randomized to daily acyclovir. This may reduce the generalizability of our findings, as little is known about the interactions or the effect on the host immune response between HSV-2 and HPV infection and between acyclovir and antiretroviral therapy. However, HSV-2 infection among HIV-positive individuals is quite common [35] and we observed no difference in the association between ART initiation and HPV detection among women taking acyclovir or placebo. Our study design was based on short-term follow-up but the use of monthly sampling for HPV detection allowed us to examine potential variations in the pattern of HPV detection. Our multivariate analyses were limited by issues of model convergence, which are common using log-binomial models to estimate prevalence ratios [36]. However, these estimates are preferable over logistic regression since odds ratios tend to overestimate risk when the outcome is common [37]. We constructed several multivariate models that included variations of the original variables found to be significant in univariate analyses and all adjusted models indicated that ART regimen, pre-ART CD4 count, and early vs. late pre-ART time period were associated with HPV detection (see Table S1). Although we did not collect data on recent sexual behavior, confounding by changes in sexual risk behavior is likely minimal since the time frame was relatively short, and we did not observe a post-ART increase in HPV detection to suggest HPV acquisition via increased sexual activity. A study of HPV and ART initiation in the Women's Interagency HIV Study (WIHS) found that adjusting for sexual behavior had no impact on their findings [13].

In conclusion, we found no impact of ART on HPV detection within 6 months of treatment initiation, despite systemic immune reconstitution. HPV detection was constitutively high, over 90% for any-HPV and 69% for HR-HPV during both the pre- and post-ART periods, and in line with previous estimates from HIV-infected women in Uganda [38]. Although current ART guidelines in many countries promote biannual cervical cancer screening in the first year after HIV diagnosis and annually thereafter, our data highlight the importance of continued and consistent screening, even after ART-initiation and immune reconstitution. Long-term surveillance data on older HIV-positive individuals will be important to determine the population-level effect of ART on cervical precancer and cancer in light of the increased cumulative burden of HPV infection due to increases in life expectancy associated with antiretroviral therapy.

## Supporting Information

Table S1 Sensitivity analysis of prevalence ratios using alternative partially adjusted log-binomial random effects models. Abbreviations: human papillomavirus (HPV); number (N), percent (%); prevalence ratio (PR); confidence interval (CI); antiretroviral therapy (ART). ^a^In unadjusted models, four-level categorical age, continuous pre-ART CD4 count, three-level ART regimen, and four-level ART initiation were significantly associated with HPV DNA detection. Log-binomial regression models would not converge for fully adjusted models. In order to better explore associations, in alternative model #1, we mutually adjust for binary age, continuous pre-ART CD4 count, three-level ART regimen, and three-level ART initiation. In alternative model #2, we mutually adjust for continuous pre-ART CD4 count, three-level ART regimen, and four-level ART initiation. ^b^Early refers to the first three months in each period and late refers to the fourth through sixth months in each period.
(DOCX)
