# Supplementary material for: Frequent Detection of HPV before and after Initiation of Antiretroviral Therapy among HIV/HSV-2 Co-Infected Women in Uganda
Source: PLoS One. 2013 Jan 29;8(1):e55383. doi: 10.1371/journal.pone.0055383 (PMC3558485; doi:10.1371/journal.pone.0055383)
Supplement: Table S1 — Sensitivity analysis of prevalence ratios using alternative partially adjusted log-binomial random effects models. Abbreviations: human papillomavirus (HPV); number (N), percent (%); prevalence ratio (PR); confidence interval (CI); antiretroviral therapy (ART). aIn unadjusted models, four-level categorical age, continuous pre-ART CD4 count, three-level ART regimen, and four-level ART initiation were significantly associated with HPV DNA detection. Log-binomial regression models would not converge for fully adjusted models. In order to better explore associations, in alternative model #1, we mutually adjust for binary age, continuous pre-ART CD4 count, three-level ART regimen, and three-level ART initiation. In alternative model #2, we mutually adjust for continuous pre-ART CD4 count, three-level ART regimen, and four-level ART initiation. bEarly refers to the first three months in each period and late refers to the fourth through sixth months in each period. (DOCX) [file pone.0055383.s001.docx]

Table S1. Sensitivity analysis of prevalence ratios using alternative partially adjusted log-binomial random effects models

|  | **Adjusted model #1 PR (95% CI)** ^a^ |
| --- | --- |
| **Age** |  |
| 20-39 years | 1 |
| 40+ years | 0.87 (0.73, 1.03) |
| **Pre-ART CD4 count** |  |
| per 100 count increase | 0.94 (0.89, 0.99) |
| **ART regimen** |  |
| None (pre-ART) | 1 |
| Other | 1.07 (1.02, 1.12) |
| AZT/3TC/NVP | 1.02 (0.96, 1.09) |
| **ART initiation^b^** |  |
| Early pre-ART | 1 |
| Late pre-ART | 1.07 (1.02, 1.13) |
| Post-ART | 1.00 (0.99, 1.00) |
|  |  |
|  | **Adjusted model #2 PR (95% CI)** ^a^ |
| **Pre-ART CD4 count** |  |
| per 100 count increase | 0.90 (0.82, 0.97) |
| **ART regimen** |  |
| None (pre-ART) | 1 |
| Other | 1.10 (1.04, 1.16) |
| AZT/3TC/NVP | 1.03 (0.97, 1.10) |
| **ART initiation^b^** |  |
| Early pre-ART | 1 |
| Late pre-ART | 1.07 (1.02, 1.13) |
| Early post-ART | 0.98 (0.96, 1.00) |
| Late post-ART | 1.00 (0.99, 1.00) |

Abbreviations: human papillomavirus (HPV); number (N), percent (%); prevalence ratio (PR); confidence interval (CI); antiretroviral therapy (ART)

^a^In unadjusted models, four-level categorical age, continuous pre-ART CD4 count, three-level ART regimen, and four-level ART initiation were significantly associated with HPV DNA detection. Log-binomial regression models would not converge for fully adjusted models. In order to better explore associations, in alternative model #1, we mutually adjust for binary age, continuous pre-ART CD4 count, three-level ART regimen, and three-level ART initiation. In alternative model #2, we mutually adjust for continuous pre-ART CD4 count, three-level ART regimen, and four-level ART initiation.

^b^Early refers to the first three months in each period and late refers to the fourth through sixth months in each period.
